# Hemolysis Induced by Pulsed‐Field Ablation of Atrial Arrhythmias: A Comparative Analysis of Current Systems

**DOI:** 10.1111/jce.70049

**Published:** 2025-08-07

**Authors:** Johannes Bruss, Thomas Kueffer, Hildegard Tanner, Fabian Noti, Andreas Haeberlin, Gregor Thalmann, Nikola Asenov Kozhuharov, Boldizsar Kovacs, Valon Spahiu, Claudia Herrera Siklody, Tobias Reichlin, Laurent Roten

**Affiliations:** ^1^ Department of Cardiology, Inselspital Bern University Hospital, University of Bern Bern Switzerland; ^2^ Sitem Center for Translational Medicine and Biomedical Entrepreneurship University of Bern Bern Switzerland

**Keywords:** atrial arrhythmia, atrial fibrillation, atrial flutter, hemolysis, lattice tip focal catheter, pentaspline catheter, pulmonary vein isolation, pulsed‐field ablation, variable loop circular catheter

## Abstract

**Introduction:**

Pulsed‐field ablation (PFA) is an emerging technology associated with dose‐dependent hemolysis as a recently recognized side effect. This study aimed to compare hemolysis levels and assess dose‐dependency across three PFA systems: a pentaspline catheter (PSC), a lattice‐tip focal catheter (LTFC), and a variable loop circular catheter (VLCC).

**Methods:**

Patients treated for atrial arrhythmias with the LTFC (*n* = 29) or the VLCC (*n* = 30) were included from a prospective registry. A matched cohort of patients treated with the PSC (*n* = 28) was recruited from the same registry. Creatinine levels and markers of hemolysis were measured pre‐ablation and 1 day postablation.

**Results:**

Haptoglobin levels decreased significantly more with the PSC and VLCC compared to the LTFC (−0.65 [−0.76, −0.49] g/L; −0.56 [−0.78, −0.43] g/L, −0.21 [−0.32, −0.1] g/L, respectively; *p* < 0.001 for both). Per‐application decreases in haptoglobin also differed (−17.5 [−20.38, −13.58] mg/L, −24.35 [−36.36, −17.92] mg/L, −3.61 [−5.98, −2.13] mg/L, respectively; *p* < 0.001 for both). There was no significant difference in haptoglobin decrease between the PSC and VLCC per procedure (*p* = 1.0). Haptoglobin decrease per application was significantly larger with the VLCC compared to the PSC (*p* = 0.0048). Per procedure LDH increase followed a similar trend (49 [18, 81.25] U/L; 14 [6, 60] U/L; 13 [−4, 46] U/L; respectively; *p* = 0.037). No hemolysis‐related complications were observed.

**Conclusions:**

Hemolysis levels vary significantly among PFA platforms. Focal PFA catheters induce less hemolysis per procedure and application compared to large‐footprint catheters.

## Graphical abstract

Real world, clinical comparison of hemolysis induced by different pulsed‐field ablation (PFA) platforms. Focal PFA catheters induce less hemolysis per procedure and application compared to large‐footprint catheters.

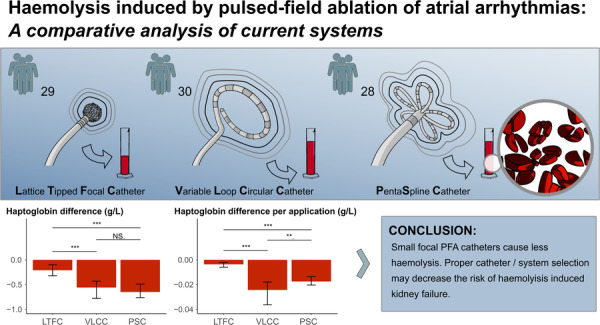

**Abbreviations**
eGFR: estimated glomerular filtration rate
LDH: lactate dehydrogenase
LTFC: lattice‐tip focal catheter
PFA: pulsed‐field ablation
PVI: pulmonary vein isolation
PSC: pentaspline catheter
VLCC: variable loop circular catheter

## 1 Introduction

In recent years, pulsed‐field ablation (PFA) has gained widespread adoption for pulmonary vein isolation (PVI) due to its efficiency and favorable safety profile compared to thermal ablation technologies [1, 2, 3, 4]. Beyond PVI, clinicians have increasingly utilized PFA to target additional areas, such as the posterior wall [5]. The pentaspline catheter (PSC) was the first commercially available system for performing PFA, approved in 2021 in Europe. However, additional PFA systems with different catheter designs, including a lattice‐tip large‐area focal catheter (LTFC), a variable‐loop circular catheter (VLCC), and others have obtained FDA approval in recent years.

One recognized side effect of PFA is hemolysis, attributed to the susceptibility of erythrocytes to the intense electric fields generated by the catheters. Although rare, a small number of acute kidney injury cases, likely caused by hemolysis, have been reported in several studies [1, 6, 7, 8, 9, 10, 11, 12, 13]. These reports to date mainly involved the pentaspline catheter. A correlation between the number of PFA applications, field strength, tissue contact and hemolysis has been observed in multiple in‐vivo as well as in‐vitro studies [14, 15]. Since different PFA systems generate electric fields of varying sizes, intensities, and configurations, differences in the degree of hemolysis per application are expected. This retrospective, single‐center study aims to investigate and compare the extent of hemolysis induced by three commercially available PFA systems.

## 2 Methods

### 2.1 Study Population

Patients with AF undergoing PVI and ablation for other atrial arrhythmia at our center are prospectively enrolled into a registry. From this registry, all consecutive patients undergoing a procedure with either the LTFC (*n* = 29) or the VLCC (*n* = 30) between April 2024 and December 2024 were included in the study. Additionally, a matched cohort of 28 patients who underwent a procedure using the PSC was selected from the same registry. Matching was performed based on age, sex, type of arrhythmia and atrial fibrillation subtype if applicable, treatment of atrial flutter during the procedure, and procedure type (primary intervention or redo). The registry was approved by the local ethics committee and conducted in accordance with the principles outlined in the Declaration of Helsinki. All patients were informed in writing about the usage of the collected data and gave written informed consent before inclusion. The authors had full access to the data set and assume full responsibility for its accuracy.

### 2.2 Ablation Procedures

Before the ablation procedure intracardiac thrombi were excluded and atrial anatomy assessed by cardiac computed tomography angiography and/or transoesophageal echocardiography. Deep conscious sedation with propofol, midazolam and fentanyl was administered following a physician‐supervised, nurse‐implemented protocol [16]. Patients at high risk for sedation‐related complications as well as early patients treated with the LTFC were managed with general anesthesia. Left atrial access was achieved via fluoroscopy‐guided transseptal puncture. Heparin was administered to maintain an activated clotting time of over 350 s.

3D mapping was performed as necessary and the specific arrhythmia targeted. In first procedures for treatment of atrial fibrillation, PVI was conducted following standard ablation protocols as recommended by the manufacturer. In redo procedures, PVI was first confirmed, and pulmonary veins were re‐isolated as needed. Additional ablation targets were determined at the discretion of the treating physician and typically included the ablation of specific arrhythmias or isolation of the left atrial posterior wall. For the procedures, we used one of the following PFA platforms:

1. A lattice tip focal catheter (Sphere‐9, Medtronic, Minneapolis, MN, USA)
2. A variable loop circular catheter (Varipulse, Biosense Webster, Irvine, CA, USA)
3. A pentaspline catheter (Farawave, Boston Scientific, Menlo Park, CU, USA)

No pre‐ or post‐procedural fluids were given to prevent hemolysis‐induced kidney failure.

### 2.3 Laboratory Analysis

Routine bloodwork was conducted 1 day before or at the procedure date before the procedure and on the day after the procedure, including measurements of creatinine, lactate dehydrogenase (LDH), haptoglobin, direct and indirect bilirubin. The analysis was performed in the hospital's clinical laboratory using standard diagnostic assays. Glomerular filtration rates were calculated from serum creatinine using the 2021 modification of the CKD‐EPI formula [17].

### 2.4 Statistical Analysis

The matched cohort of patients undergoing PFA using a PSC was created from the aforementioned registry using the TriMatch R‐package [18]. This algorithm creates matched triplets of observations by minimizing the distance derived from propensity scores between each combination of observations as much as possible with the given cohorts (PLCC vs. PSC, PLCC vs. LTFC, LTFC vs. PSC). Propensity scores were estimated using logistic regression. To assess the influence of the remaining variance after matching, ANCOVA was performed with the PFA system, the number of PFA applications and the matching variables as additional as covariates.

Categorical variables were reported as counts (percentage), continuous variables as medians with interquartile ranges (1st quartile, 3rd quartile) or means ± standard deviations. Fisher's exact test, the Kruskal–Wallis rank sum test, or the pairwise Wilcoxon test were applied as appropriate. Correlation was tested with the Pearson's product‐moment correlation. For pairwise comparisons, *p*‐values were adjusted using Bonferroni correction. All statistical analysis was performed in R [19].

## 3 Results

### 3.1 Patient and Procedural Characteristics

A total of 87 patients were included in the study (median age 68 years; 29% female). The LTFC was used in 29 cases, the VLCC in 30, and the PSC in 28. Baseline patient characteristics are summarized in Table 1 and procedural characteristics in Table 2. Both left and right atrial flutter as well as redo PVI were more frequently targeted with the LTFC system. A graphical representation of all PFA targets is shown in the Supporting Information: Figure 1. The overall procedure duration was longest with the LTFC system, followed by the VLCC and PSC systems, with median durations of 120, 105, and 78 min, respectively. Use of the LTFC system required the highest number of PFA applications, followed by the PSC and VLCC, with median counts of 50, 36, and 22, respectively.

**Table 1:** Baseline patient characteristics.

	Total (*n* = 87)	LTFC (*n* = 29)	VLCC (*n* = 30)	PSC (*n* = 28)	*p*‐value
Women (%)	25 (29)	9 (31)	8 (27)	8 (29)	0.933
Age (years)	68.2 (59.8, 76.77)	65.7 (59.1, 73.3)	76.2 (60.9, 78.6)	67.9 (60.2, 74.8)	0.229
Weight (kg)	85 (73, 97)	91 (75, 103)	76.5 (66, 92)	84.5 (76, 94)	0.069
Height (cm)	176 (168, 183)	178 (168, 185.25)	176 (166.75, 179.5)	175 (167, 183)	0.72
LVEF (%)	55 (45, 60)	55 (45, 60)	55 (45, 60)	55 (46.25, 60)	0.811
LAVI (mL/m^2^)	41.5 (35, 47)	46.5 (36.5, 48)	39 (35, 46)	43 (34, 49)	0.439
Ischemic heart disease	8 (9)	4 (14)	2 (7)	2 (7)	0.648
Valvular heart disease	12 (14)	3 (10)	4 (13)	5 (18)	0.686
Hypertensive heart disease	15 (17)	3 (10)	8 (27)	4 (14)	0.255
Diabetes	11 (13)	3 (10)	2 (7)	6 (21)	0.251
Prior stroke	6 (7)	1 (3)	0 (0)	5 (18)	0.009
CHA_2_DS_2_‐VA Score	2 (1, 3)	2 (1, 2)	2 (1, 3)	2 (1, 3)	0.014
0	15 (17)	7 (25)	4 (13)	4 (14)	
1	15 (17)	6 (21)	4 (13)	5 (18)	
2	27 (31)	10 (36)	10 (33)	7 (25)	
3	21 (24)	3 (11)	12 (40)	6 (21)	
4	6 (7)	0 (0)	0 (0)	6 (21)	
5	2 (2)	2 (7)	0 (0)	0 (0)	
Atrial fibrillation (%)	78 (90)	22 (76)	28 (93)	28 (100)	0.011
Paroxysmal	36 (41)	8 (28)	17 (57)	11 (39)	
Persistent	42 (48)	14 (48)	11 (37)	17 (61)	
Atrial flutter (%)	34 (39)	21 (72)	3 (10)	10 (36)	< 0.001

*Note:* Shown are counts with percentages or medians with interquartile ranges (1st quartile, 3rd quartile).

Abbreviations: LAVI, left atrial volume index; LTFC, lattice tip focal catheter; LVEF, left ventricular ejection fraction; PSC, pentaspline catheter; VLCC, variable loop circular catheter.

**Table 2:** Procedural characteristics.

	Total (*n* = 87)	LTFC (*n* = 29)	VLCC (*n* = 30)	PSC (*n* = 28)	*p*‐value
PVI performed					0.015
Primary PVI (% of all procedures)	36 (41)	8 (28)	16 (53)	12 (43)	
Redo PVI (% of all procedures)	42 (48)	14 (48)	12 (40)	16 (57)	
Right atrial flutter ablation (% of all procedures)	5 (6)	5 (17)	0 (0)	0 (0)	0.004
Left atrial flutter ablation (% of all procedures)	16 (18)	13 (45)	1 (3)	2 (7)	< 0.001
Total PFA applications (n)	36 (22.5, 47)	50 (41, 64)	21.5 (19, 25)	36 (29.75, 44)	< 0.001
Total RFA applications (n)	16 (10,30)	16 (10,30)	—	—	—
Fluoroscopy time (min)	7 (5, 11.5)	7 (4, 10)	6 (4, 6.75)	12.5 (8.75, 17.25)	< 0.001
Procedure duration (min)	105 (78, 123)	120 (87, 150)	105 (85, 122)	78 (59.75, 107)	< 0.001
3D‐Mapping (%)	67 (77)	29 (100)	30 (100)	8 (29)	< 0.001

*Note:* Shown are counts with percentages or medians with interquartile ranges (1st quartile, 3rd quartile).

Abbreviations: LTFC, lattice tip focal catheter; PFA, pulsed‐field ablation; PSC, pentaspline catheter; PVI, pulmonary vein isolation; RFA, radiofrequency ablation; VLCC, variable loop circular catheter.

### 3.2 Pre‐ and Post‐Procedural Bloodwork

Results of pre‐ and post‐procedural bloodwork are presented in Supporting Information: Tables 1 and 3, respectively. There were no significant differences in the preprocedural hemolysis markers and renal function among the groups. There was also no significant difference in the interval between the intervention and the postprocedural blood draw between the groups (LTFC: 17.2 ± 2.8 h; VLCC 16.9 ± 2.8 h; PSC 16.9 ± 2.7 h, *p* = 0.86).

**Table 3:** Post‐procedural bloodwork.

	Total (*n* = 87)	LTFC (*n* = 29)	VLCC (*n* = 30)	PSC (*n* = 28)	*p*‐value
Creatinine (µmol/L)	84 (74, 101)	84 (72, 101)	84 (74.5, 94)	87.5 (75.75, 103.25)	0.678
Difference (µmol/L)	0 (‐6, 6)	0 (−5, 2)	−2.5 (−6.75, 4.75)	3 (−1.25, 6.25)	0.228
eGFR (mL/min)	85.93 (70.83, 94.35)	87.6 (72.39, 97.13)	86.15 (78.24, 92.47)	85.53 (68.19, 95.02)	0.851
Difference (mL/min)	0 (−3.37, 4.39)	0 (−2.55, 4.76)	1.09 (−2.99, 4.58)	−1.3 (−5.18, 0.89)	0.226
Direct bilirubin (µmol/L)	6 (4.1, 8.4)	5.9 (4.5, 9)	6.15 (4.1, 7.88)	6.05 (4, 8.55)	0.833
Difference (µmol/L)	1.9 (1, 3.08)	1.6 (1, 2.7)	2.1 (0.6, 3)	2.2 (1, 3.2)	0.75
Total bilirubin (µmol/L)	16.7 (10.6, 22.65)	16.8 (11.1, 27.5)	16.2 (11.03, 21.2)	16.05 (9.97, 23.25)	0.77
Difference (µmol/L)	4.55 (1.3, 8.85)	4 (1.1, 9.3)	4.5 (1.2, 7.5)	6.65 (3.35, 10.27)	0.231
LDH (U/L)	245 (215, 285)	241 (202, 277)	244 (207.75, 270.75)	257.5 (238.75, 310.25)	0.056
Difference (U/L)	26 (6, 67)	13 (−4, 46)	14 (6, 60)	49 (18, 81.25)	0.011
Haptoglobin (g/L)	0.68 (0.32, 1.01)	1.01 (0.8, 1.6)	0.44 (0.2, 0.72)	0.58 (0.3, 0.82)	< 0.001
Difference (g/L)	−0.49 (−0.69, −0.26)	−0.21 (−0.32, −0.1)	−0.56 (−0.78, −0.43)	−0.65 (−0.76, −0.49)	< 0.001
Per application (mg/L)	−15.01 (−22.6, −6.08)	−3.61 (−5.98, −2.13)	−24.35 (−36.36, −17.92)	−17.5 (−20.38, −13.58)	< 0.001

*Note:* Shown are medians with interquartile ranges (1st quantile, 3rd quantile). Differences were calculated by subtracting preprocedural measurements from postprocedural measurements. For haptoglobin, the difference per PFA application is also provided, calculated by dividing the pre‐to‐post difference by the total number of PFA applications.

Abbreviations: EGFR, estimated glomerular filtration rate; LDH lactate dehydrogenase; LTFC, lattice tip focal catheter; PSC, pentaspline catheter; VLCC, variable loop circular catheter.

### 3.3 Haptoglobin Levels

**Figure 1:**
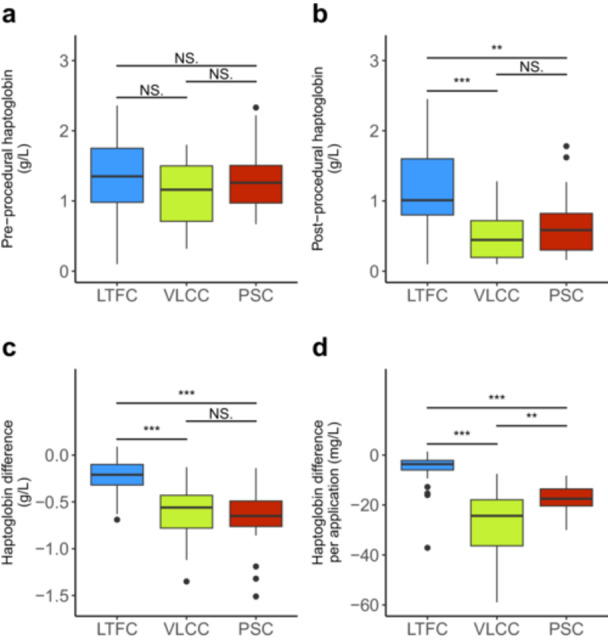
Boxplots showing haptoglobin levels according to the PFA system used. (a) Preprocedural haptoglobin levels. (b) Postprocedural haptoglobin levels. (c) Absolute (g/L) pre‐to‐post‐procedural differences in haptoglobin. (d) Absolute (mg/L) pre‐to‐post‐procedural differences in haptoglobin, normalized per PFA application. LTFC, lattice tip focal catheter; PFA, pulsed‐field ablation; PSC, pentaspline catheter; VLCC, variable loop circular catheter. (NS., not significant; *p < 0.05; **p < 0.01; ***p < 0.001).

**Figure 2:**
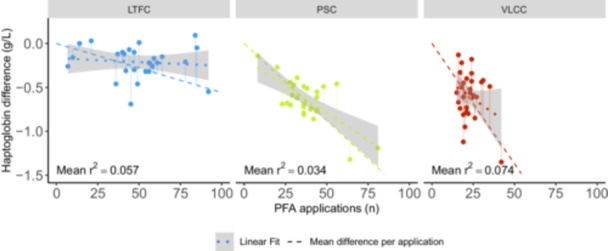
Correlation of haptoglobin levels with the number of PFA applications for each PFA system. A linear regression fit is represented by the dotted line, with the 95% confidence interval shaded in gray. The mean haptoglobin difference per PFA application observed between pre‐ and postprocedural measurements is used as the slope of the dashed line. The mean residuals squared to this simple estimate are displayed within each panel. LTFC, lattice tip focal catheter; PFA, pulsed‐field ablation; PSC, pentaspline catheter; VLCC, variable loop circular catheter.

Post‐procedural haptoglobin levels were significantly lower in the VLCC and PSC groups compared to the LTFC group (*p* < 0.001 and *p* < 0.01, respectively), but no significant difference was observed between the VLCC and PSC groups (*p* = 0.79; Figure 1; Supporting Information: Figure 2; Table 3). Accordingly, the pre‐to‐post difference of haptoglobin levels were greater in the VLCC and PSC groups compared to the LTFC group (*p* < 0.001 and *p* < 0.001, respectively; Figure 1; Table 3), but no significant difference was found between the VLCC and PSC groups (*p* = 1). Normalized for the number of PFA applications in each group, the decrease of haptoglobin levels per application was lowest for the LTFC system, and highest for the VLCC system, with a significant difference between all three system (LTFC vs. PSC *p* < 0.001; LTFC vs. VLCC *p* < 0.001; PSC vs. VLCC *p* < 0.005). Linear regression models fitted to the pre‐to‐post difference of haptoglobin levels against the number of PFA applications indicated a decrease in serum haptoglobin levels with increasing number of applications across all PFA system (Figure 2). However, this correlation was statistically significant only for the PSC system (*p* < 0.001), but not for the LTFC (*p* = 0.222) or VLCC (*p* = 0.126) systems. ANCOVA showed no significant influence of either age, sex, type of AF or treatment of atrial flutter on haptoglobin difference and haptoglobin difference per application. PVI redoes showed a minimally significant influence on haptoglobin per application (*p* = 0.0445), the number of PFA applications was significant on haptoglobin difference per procedure (*p* < 0.001; Supporting Information: Table 2, Supporting Information: Figure 3).

### 3.4 Other Hemolysis Parameters

**Figure 3:**
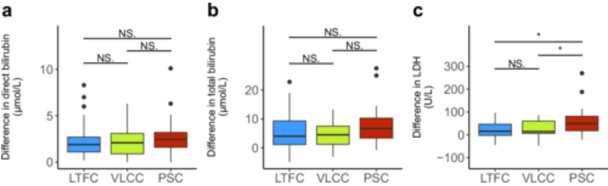
Boxplots illustrating absolute (µmol/L or U/L) pre‐to‐post‐procedural differences of direct bilirubin (a), total bilirubin (b), and LDH (c) measurements according to the PFA system used. LDH, lactate dehydrogenase; LTFC, lattice tip focal catheter; PSC, pentaspline catheter; VLCC, variable loop circular catheter. (NS., not significant; *p < 0.05; **p < 0.01; ***p < 0.001).

No statistical difference in preprocedural direct bilirubin, total bilirubin or LDH was observed (Supporting Information: Figures 2 and 4). Neither direct bilirubin nor total bilirubin showed a statistically significant pre‐to‐post difference among the three groups (Figure 3). However, the absolute pre‐to‐post difference of LDH was significantly greater following PFA with the PSC system compared to the LTFC (*p* = 0.024) or VLCC (*p* = 0.037) system.

### 3.5 Hemolysis Related Kidney Injury

**Figure 4:**
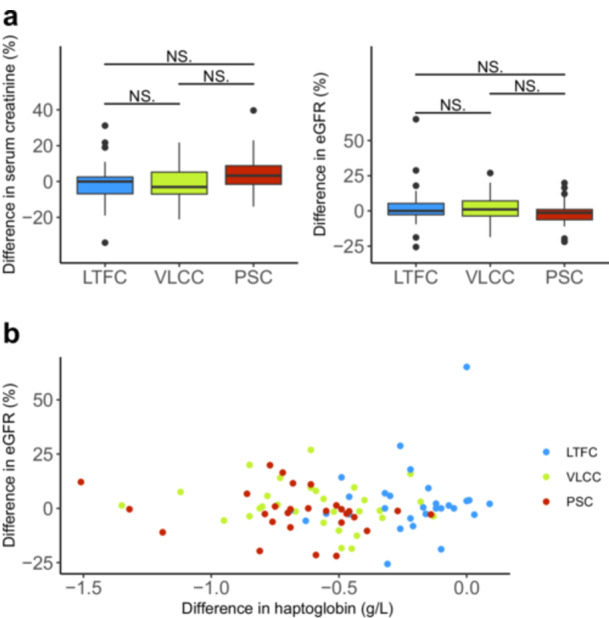
(a) Boxplots showing the relative (%) pre‐to‐post‐procedural differences in serum creatinine and estimated glomerular filtration rate (eGFR) measurements according to the PFA system used. (b) Dot plot illustrating the relative (%) pre‐to‐post‐procedural difference in eGFR plotted against the absolute (g/L) pre‐to‐post‐procedural difference in haptoglobin, categorized by the PFA system used. eGFR, estimated glomerular filtration rate; LTFC, lattice tip focal catheter; PSC, pentaspline catheter; PFA, pulsed‐field ablation; VLCC, variable loop circular catheter. (NS., not significant; *p < 0.05; **p < 0.01; ***p < 0.001).

No cases of acute kidney injury, defined as an increase in serum creatinine exceeding 1.5 times the baseline value or above 26.5 µmol/L as outlined by the KDIGO criteria [20] was observed in the study population. Additionally, no significant impact of PFA on renal function was found among the three groups (Figure 4; Supporting Information: Figure 5). Furthermore, no significant correlation was observed between changes in haptoglobin levels and eGFR (*p* = 0.76).

## 4 Discussion

Our main findings are:

1. Hemolysis was observed with all three PFA systems investigated in this study.
2. Hemolysis was more pronounced with the PSC and VLCC systems compared to the LTFC, both at a procedural and a per application level.
3. Within the number of PFA applications used in our study, no clinically meaningful hemolysis‐related adverse effects were observed with any of the investigated PFA systems.

After its market introduction in 2021, two cases of acute kidney injury potentially linked to severe intravascular hemolysis following PFA were reported in 2023 [6]. In both cases, the PSC system was used and over 100 PFA applications were delivered, which was significantly more compared to the standard protocol of 32 applications (8 per vein) tested and subsequently approved in the IDE trials [21, 22, 23]. In the same manuscript, the authors reported an inverse relationship between haptoglobin levels—a laboratory marker for intravascular hemolysis—and the number of PFA applications. This dose‐dependent effect has been observed not only in human studies but also in in vitro and animal models [13, 14, 15]. Moreover, subsequent investigations demonstrated that this phenomenon is not induced by thermal ablation modalities such as radiofrequency or cryoballoon ablation [11, 24].

The clinical relevance of PFA‐induced hemolysis was further supported by a large multicentre study, which reported an incidence of hemolysis‐induced acute kidney failure necessitating dialysis in 5 out of over 17,000 patients (0.03%) [1]. To date, only one study has compared different PFA systems regarding hemolysis, specifically the PSC and VLCC, finding no significant differences among them [8]. In addition to these large form‐factor catheters, which are optimized for PVI, a large‐area focal PFA catheter with a lattice‐tip design—the LTFC—has been introduced. Since the extent of red blood cell exposure to the electrical field is determined by the catheter size, electrode configuration, field strength, and the pulse protocol, varying degrees of hemolysis are expected. Accordingly, larger catheter designs tend to induce more hemolysis per application, all else being equal [25]. Consistent with this hypothesis, we observed less hemolysis with the LTFC compared to the VLCC and PSC, as indicated by a smaller decrease in haptoglobin levels. On the other hand, LDH increased most markedly in the PSC group but to a lesser extent in the VLCC and LTFC groups. As LDH is a general marker of cell death, both hemolysis and myocardial tissue destruction caused by ablation contribute to its elevation. The distinct rise in the PSC group may also result from more extensive myocardial tissue damage induced by this catheter. Differences in direct and total bilirubin can assist in assessing hemolysis, but they are less specific and sensitive than a decrease in haptoglobin. The small size of our patient populations likely limits the ability to detect differences in this less sensitive marker.

When adjusting for the number of PFA applications, thereby accounting for the differences of the ablation procedures, the difference between the systems became more pronounced, with a significant difference also emerging between the VLCC and PSC systems. The VLCC system resulted in higher hemolysis per application, although this effect was counterbalanced at the procedural level by requiring fewer applications for successful PVI compared to the PSC. De Smet et al. previously reported a comparable reduction in haptoglobin levels, with both systems demonstrating a similar level of hemolysis at the procedural level, whether using the VLCC or the PSC [8].

Additionally, we confirmed the dose‐dependent relationship between hemolysis and the number of applications observed in previous studies for single‐shot catheters and extended this finding to focal ablation catheters. In all three systems, haptoglobin levels declined with increasing numbers of applications. However, this correlation was statistically significant only for the PSC system, likely due to the small sample size and the relatively low hemolysis induced by the LTFC. Previous studies have clearly shown that hemolysis is a class effect of PFA, but the degree of hemolysis is determined by the electric field intensity, which varies among catheters and is not publicly disclosed by manufacturers. Additionally, factors such as the number of PFA applications and catheter‐tissue contact are also important determinants [9]. The larger and significant effect on haptoglobin levels per application observed with the PSC, compared to the other catheters, may result from a combination of these factors.

Haptoglobin was chosen for comparison as it is a well‐established marker of mild intravascular hemolysis. Its primary limitation is the inability to assess moderate to severe hemolysis, as it is entirely depleted in such cases. In contrast, bilirubin and LDH are commonly used as complementary markers [26, 27, 28, 29]. Since PFA generally causes only minor hemolysis, haptoglobin is particularly well‐suited for detecting these small changes. In addition, haptoglobin provides greater robustness compared to other indirect hemolysis markers such as LDH or bilirubin, since these parameters can be elevated due to inflammation or myocardial damage. Importantly, the inflammation induced by catheter ablation does not affect the specificity of haptoglobin for hemolysis [30, 31]. While the analysis of free hemoglobin and haptoglobin‐bound hemoglobin could offer a more precise assessment of hemolysis severity, practical constraints in a register‐based study limit its feasibility due to the rapid elimination of these complexes through binding and renal excretion [32, 33, 34]. In vitro studies have demonstrated an inverse linear relationship between free hemoglobin and haptoglobin levels, supporting the quasi‐linear association inferred in this study [34].

No patient in our study showed an increase in serum creatinine fulfilling the definition of acute kidney injury according to the KDIGO (Kidney Disease: Improving Global Outcomes) definition of 2012 [20]. Given the low incidence of hemolysis‐induced kidney injury after PFA, as reported in the MANIFEST‐17K study, and the overall low number of PFA applications in our cases, the absence of renal failure in our relatively small sample size is expected [1].

Although rare, appropriate measures should be taken to prevent hemolysis‐induced kidney injury during PFA. In addition to minimizing the number of PFA applications and ensuring adequate fluid administration, as investigated by Mohanty et al., our findings emphasize the importance of selecting an appropriate PFA catheter based on the targeted arrhythmia [10]. Even though single shot PFA systems can be employed for the ablation of macro‐reentrant tachycardias, the LTFC, creating focal lesions akin to radiofrequency ablation, make it particularly suitable for these kind of arrhythmias [35]. If multiple focal targets require ablation, its ability to toggle between PFA and RFA in addition to the lower rate of hemolysis make it the preferred ablation catheter.

### 4.1 Limitations

This study is limited by its small sample size and its non‐randomized, single‐center, retrospective design. Hemolysis was assessed indirectly through the decrease in haptoglobin levels, as no direct measurements of free hemoglobin, quantitative blood smears, or urinary analysis of hemolysis parameters were performed.

Additionally, the arrhythmias treated and ablation targets varied between groups, reflecting real‐world clinical application of these PFA systems. As a result, direct procedural‐level comparisons of hemolysis markers may be biased; however, this was addressed by normalizing hemolysis measurements on a per‐application basis.

## 5 Conclusions

Our study confirms the anticipated impact of field size and dose dependency of PFA on hemolysis in humans. Focal PFA catheters resulted in significantly less hemolysis per application, as evidenced by a smaller decrease in haptoglobin levels, compared to large‐footprint catheters. Although no clinical adverse effects were observed, the extent of hemolysis was related to catheter design and number of applications. These findings highlight the importance of catheter selection and ablation strategy to minimize hemolysis, particularly in more extensive ablation procedures.

## Ethics Statement

The Swiss‐AF‐PVI Registry was approved by the ethics committee of the Canton of Bern, all analysis were performed as outlined in the registries approval (KEK PB_2018_00226).

## Conflicts of Interest

A. Haeberlin has received travel fees/educational grants from Medtronic, Biotronik, Abbott, and Philips/Spectranetics without impact on his personal remuneration. He serves as a proctor for Medtronic. He has received research grants from the Swiss National Science Foundation, the Swiss Innovation agency Innosuisse, the Swiss Heart Foundation, the University of Bern, the University Hospital Bern, the Velux Foundation, the Hasler Foundation, the Swiss Heart Rhythm Foundation, and the Novartis Research Foundation. He is Co‐founder and CEO of Act‐Inno AG. L. Roten has received research grants from Medtronic, the Swiss National Foundation, the Swiss Heart Foundation, the Immanuel and Ilse Straub Foundation and the Sitem Insel Support Fund, all for work outside the submitted study. He has received speaker fees/honoraria from Biosense Webster, Boston Scientific, Abbott and Medtronic. T. Reichlin Research grants from the Swiss National Science Foundation, the Swiss Heart Foundation, the sitem insel support fund, Biotronik, Boston Scientific and Medtronic, all for work outside the submitted study. He has received speaker/consulting honoraria or travel support from Abbott/SJM, Biosense‐Webster, Biotronik, Boston Scientific and Medtronic. He has received support for his institution's fellowship program from Abbott/SJM, Biosense‐Webster, Biotronik, Boston‐Scientific and Medtronic. N. Kozhuharov has received research grants from the Swiss National Science Foundation (P400PM‐194477 and P5R5PM_210856), Gottfried und Julia Bangerter‐Rhyner‐Stiftung, Freiwillige Akademische Gesellschaft, L. & Th. La Roche Stiftung and the European Society of Cardiology. F. Noti Medtronic, Abbott: Travel fees, speaker fees, educational grant; Boston Scientific, Philips Spectranetics: Travel fees, educational grant; Biotronik: Institutional grant all for work outside the submitted study. T. Kueffer research grants from the Swiss Heart Foundation for work outside the submitted study. All other authors report no conflicts of interest.

## Supporting information

Supplemental Figures JCE Revision 1 final.

## Data Availability

The data that support the findings of this study are available from the corresponding author upon reasonable request.
