# Supplementary material for: Hemolysis Induced by Pulsed‐Field Ablation of Atrial Arrhythmias: A Comparative Analysis of Current Systems
Source: J Cardiovasc Electrophysiol. 2025 Aug 7;36(10):2498–506. doi: 10.1111/jce.70049 (PMC12530677; doi:10.1111/jce.70049)
Supplement: Supplementary file 1 — Supplemental Figures JCE Revision 1 final. [file JCE-36-2498-s001.pdf]

Supplemental Figure 1:

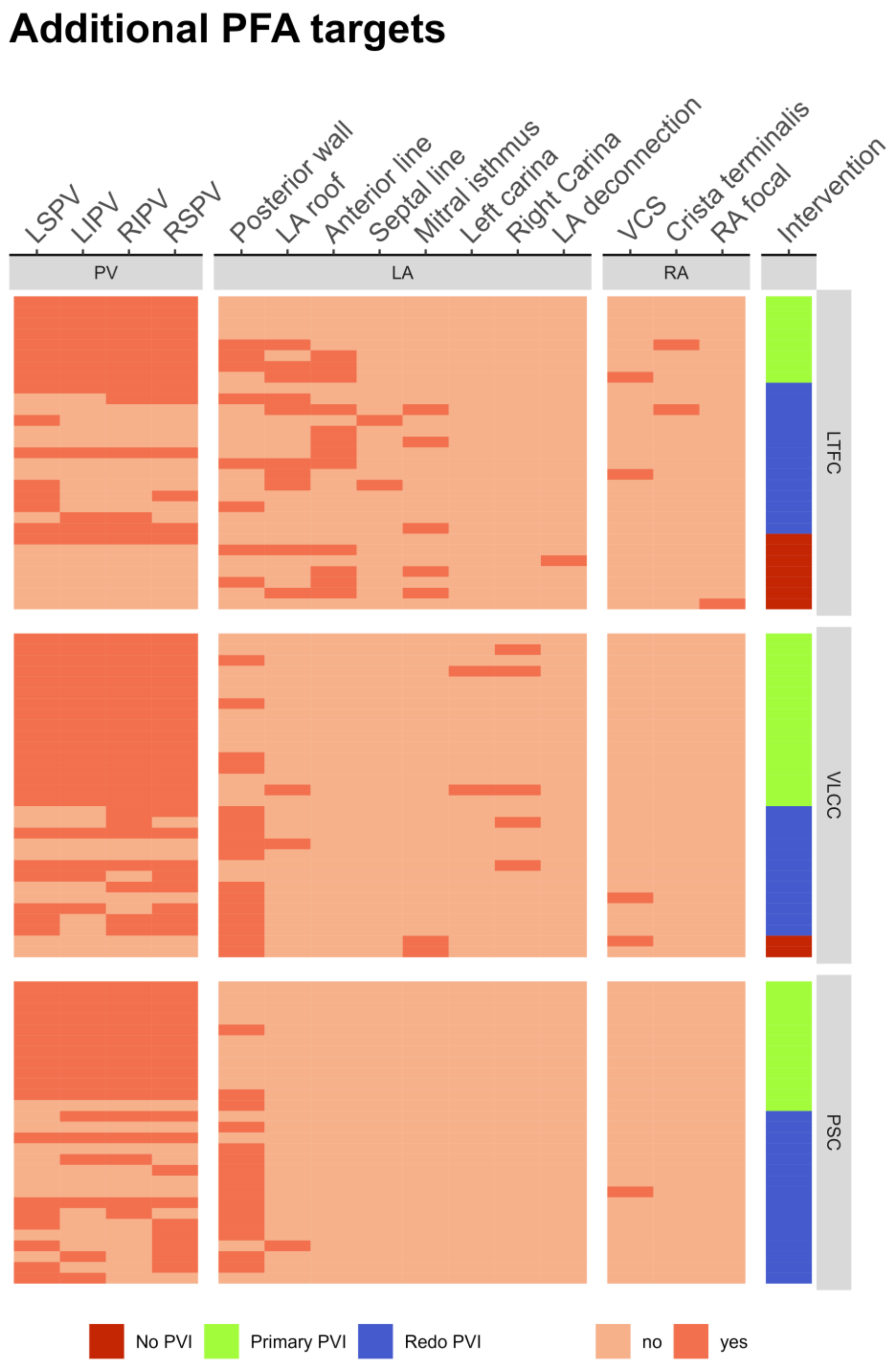

Overview of target lesions with each row representing one intervention. LA, left Atrium; LTFC, lattice tip focal catheter; PFA, pulsed-field ablation; PV, pulmonary veins; PVI, Pulmonary vein isolation; RA, right Atrium; VLCC, variable loop circular catheter; PSC, pentaspline catheter; VCS, superior vena cava.

Supplemental Table 1:

Pre-procedural bloodwork

	Total (n=87)	LTFC (n=29)	VLCC (n = 30)	PSC (n=28)	P-value
Creatinine (µmol/L)	86 (73, 97.5)	88 (73, 100)	84.5 (75, 95.75)	85.5 (72, 94.5)	0.97
eGFR (ml/min)	85.25 (73.28, 95.48)	83.47 (73.21, 97.49)	85.58 (72.51, 93.51)	85.71 (77.26, 96.26)	0.862
Hemoglobin (g/L)	140 (129, 148.5)	135 (127, 143)	143 (136.25, 151.25)	140 (127.75, 148.25)	0.145
Direct bilirubin (µmol/L)	3.65 (3, 5.77)	4.4 (3, 5.9)	3.3 (3, 5.9)	3.45 (2.75, 4.73)	0.542
Total bilirubin (µmol/L)	9.6 (7.38, 16.35)	11.3 (7.8, 16.4)	9.8 (8, 18)	9 (7.25, 13.32)	0.515
LDH (U/L)	217.5 (186.5, 243.75)	216 (180, 248)	221 (186, 246)	214.5 (193.75, 230.5)	0.931
Haptoglobin (g/L)	1.27 (0.85, 1.58)	1.35 (0.98, 1.75)	1.16 (0.71, 1.5)	1.26 (0.97, 1.5)	0.364

Shown are medians with interquartile ranges (1st quartile, 3rd quartile). EGFR, estimated glomerular filtration rate; LDH lactate dehydrogenase; LTFC, lattice tip focal catheter;; VLCC, variable loop circular catheter; PSC, pentaspline catheter.

Supplemental Figure 2:

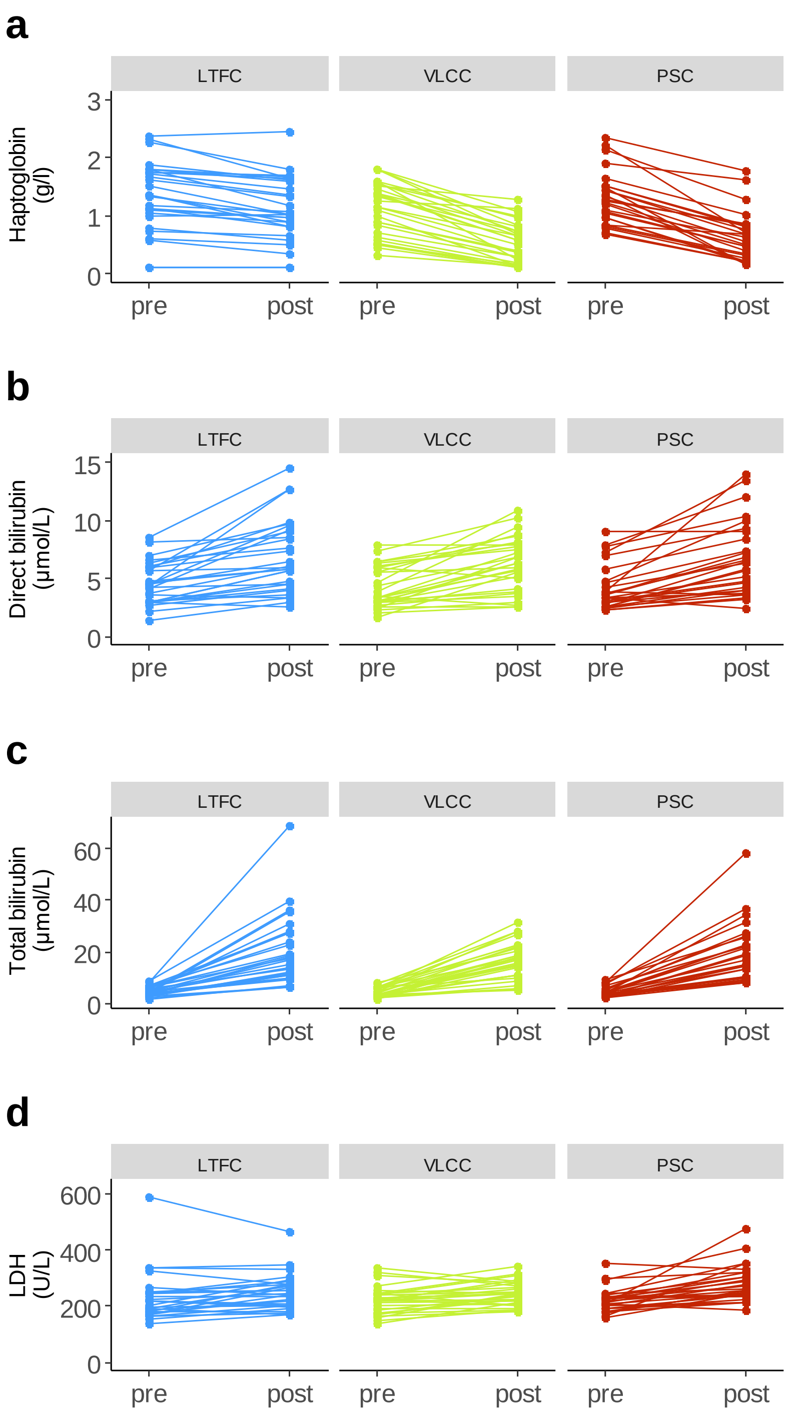

Boxplots illustrating direct bilirubin, total bilirubin, and LDH measurements according to the PFA system used. Left panels: Pre-procedural measurements. Middle panels: Post-procedural measurements. Right panels: Pre-to-post-procedural difference per PFA application. LDH, lactate dehydrogenase; LTFC, lattice tip focal catheter; VLCC, variable loop circular catheter; PSC, pentaspline catheter. (NS., not significant; *, p < 0.05; **, p < 0.01; ***, p < 0.001).

Supplemental Table 2:

ANCOVA Results for difference in haptoglobin

	Degrees of Freedom	Sum squared	F-Value	Pr (>F)	
PFA system	2	3.258	27.907	8.17E-10	***
Age	1	0.025	0.437	0.510777	
Sex	1	0.010	0.164	0.686894	
Type of AF	2	0.115	0.987	0.377548	
Atrial flutter treated	1	0.021	0.358	0.551310	
Primary / Redo PVI	1	0.005	0.084	0.772857	
PFA applications	1	0.927	15.883	0.000153	***
Residuals	76	4.437			

ANCOVA Results for difference in haptoglobin per application

	Degrees of Freedom	Sum squared	F-Value	Pr (>F)	
PFA system	2	0.006927	43.941	2.08E-13	***
Age	1	0.000126	1.602	0.210	
Sex	1	0.000016	0.201	0.655	
Type of AF	2	0.000134	0.849	0.432	
Atrial flutter treated	1	0.000000	0.001	0.975	
Primary / Redo PVI	1	0.000334	4.234	0.043	*
PFA applications	1	0.000171	2.171	0.145	
Residuals	76	0.005991			

Supplemental Figure 3:

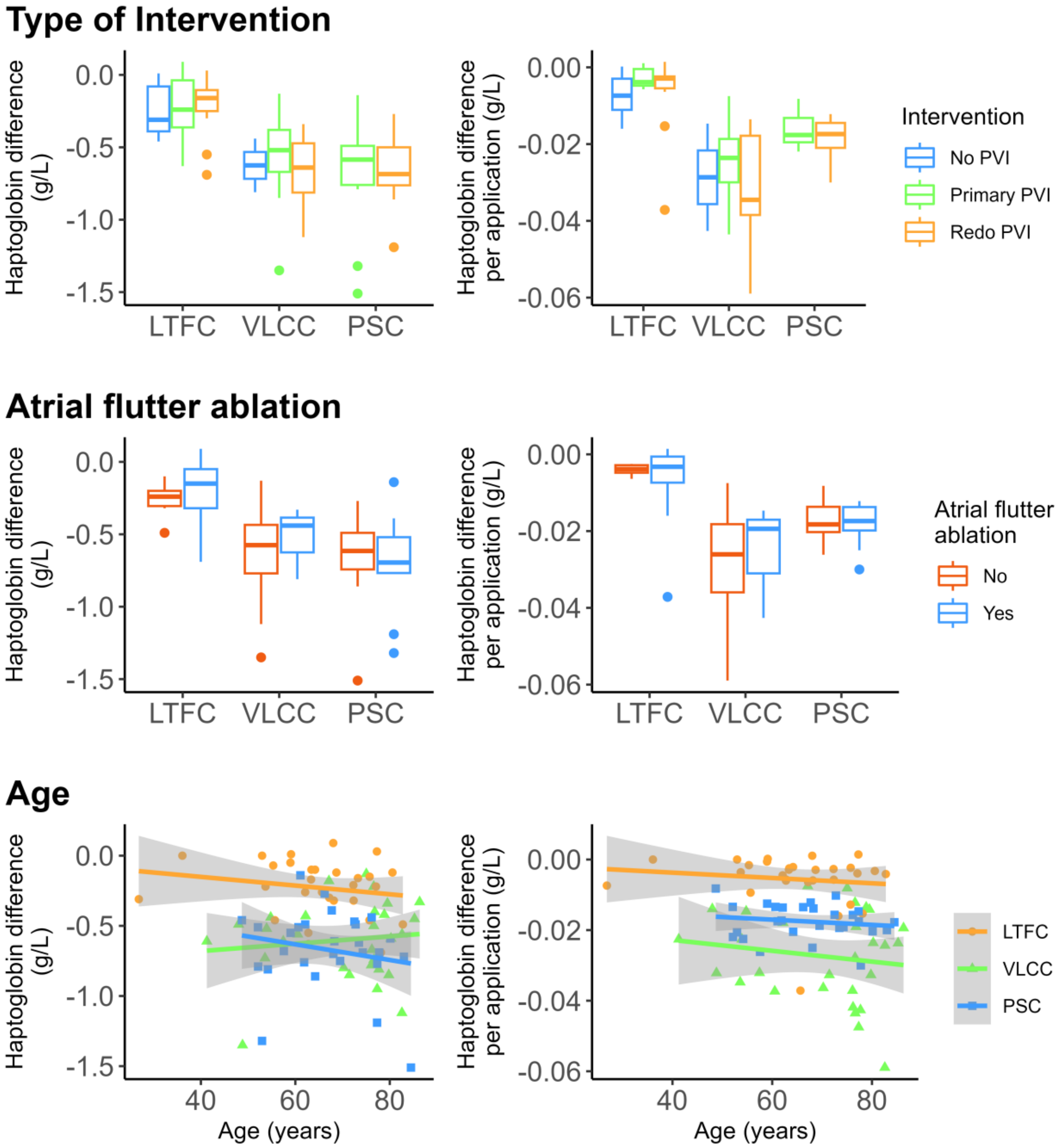

Additional boxplots and dotplots illustrating the influence of possible covariates on the difference in Haptoglobin. LTFC, lattice tip focal catheter; VLCC, variable loop circular catheter; PSC, pentaspline catheter.

Supplemental Figure 4:

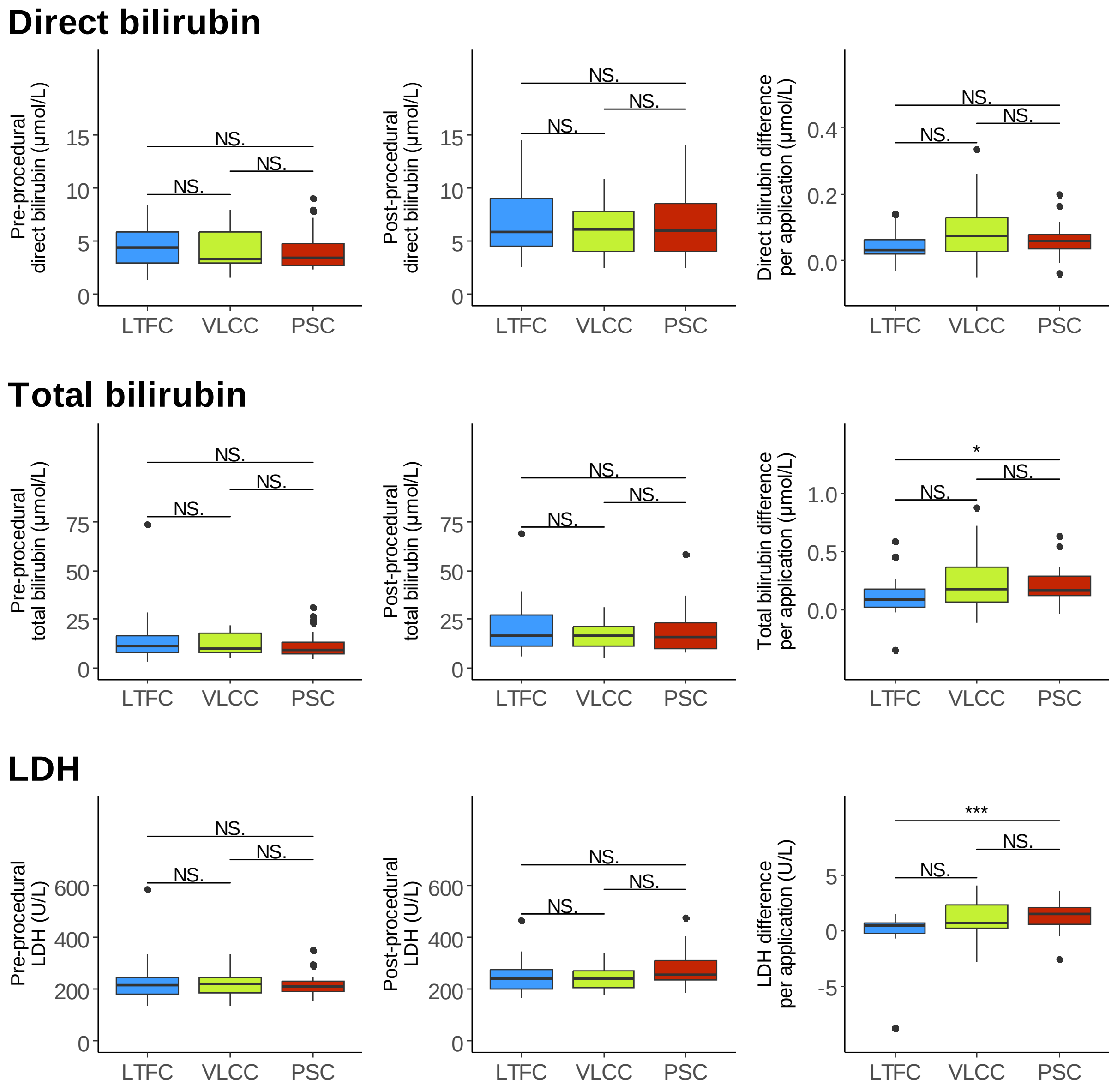

Pre- and post-procedural measurements of haemolysis parameters, with lines connecting individual patient measurements, grouped and colour-codeded by PFA system. a) Haptoglobin in g/L; b) Direct Bilirubin in µmol/L; c) Total Bilirubin in µmol/L; d) LDH in U/L. LDH, lactate dehydrogenase; LTFC, lattice tip focal catheter; PFA, pulsed-field ablation; VLCC, variable loop circular catheter; PSC, pentaspline catheter.

Supplemental Figure 5:

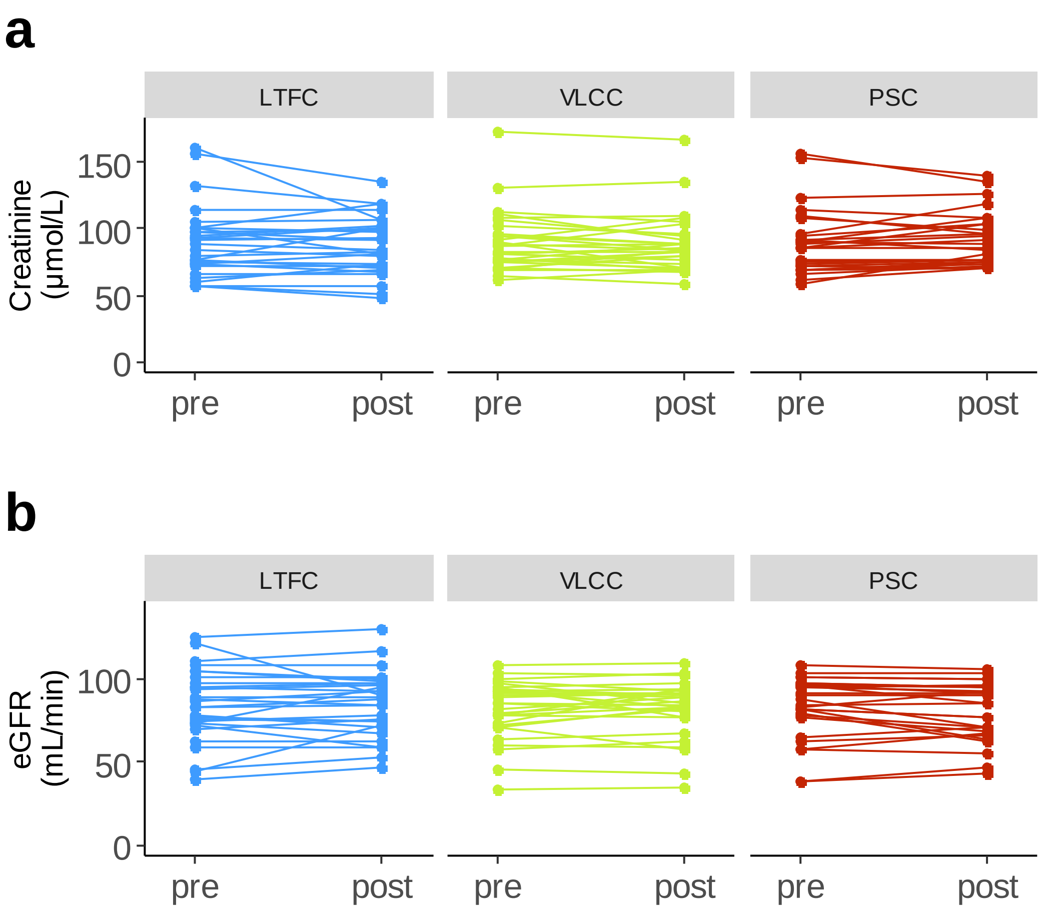

Pre- and post-procedural measurements of serum creatinine in µmol/L (a) and eGFR in ml/min (b), with lines connecting individual patient measurements, grouped and colour-coded by PFA system. eGFR, estimated glomerular filtration rate; LTFC, lattice tip focal catheter; PFA, pulsed-field ablation; VLCC, variable loop circular catheter; PSC, pentaspline catheter.
